# Cardiac structure and function in elite Native Hawaiian and Pacific Islander Rugby Football League athletes: an exploratory study

**DOI:** 10.1007/s10554-017-1285-x

**Published:** 2017-11-30

**Authors:** Christopher Johnson, Lynsey Forsythe, John Somauroo, Michael Papadakis, Keith George, David Oxborough

**Affiliations:** 1 Research Institute for Sport and Exercise Sciences, Tom Reilly Building, Liverpool John Moores University, Liverpool, L3 3AF UK; 2 Cardiology Clinical Academic Group, St Georges, University of London, London, UK

**Keywords:** Athletes heart, Left ventricle, Right ventricle, Ethnicity, Echocardiography, Strain imaging

## Abstract

The aim of this exploratory study was to define the Athletes Heart (AH) phenotype in Native Hawaiian & Pacific Islander (NH&PI) Rugby Football League (RFL) athletes. Specifically, (1) to describe conventional echocardiographic indices of left ventricle (LV) and right ventricle (RV) structure and function in NH&PI RFL players and matched RFL Caucasian controls (CC) and (2) to demonstrate LV and RV mechanics in these populations. Ethnicity is a contributory factor to the phenotypical expression of the AH. There are no data describing the cardiac phenotype in NH&PI athletes. Twenty-one male elite NH&PI RFL athletes were evaluated using conventional echocardiography and myocardial speckle tracking, allowing the assessment of global longitudinal strain (ε) and strain rate (SR); and basal, mid and global radial and circumferential ε and SR. Basal and apical rotation and twist were also assessed. Results were compared with age-matched Caucasian counterparts (CC; n = 21). LV mass [42 ± 9 versus 37 ± 4 g/(m^2.7^)], mean LV wall thickness (MWT: 9.5 ± 0.7 and 8.7 ± 0.4 mm), relative wall thickness (RWT: 0.35 ± 0.04 and 0.31 ± 0.03) and RV wall thickness (5 ± 1 and 4 ± 1 mm, all p < 0.05) were greater in NH&PI compared with CC. LV and RV cavity dimensions and standard indices of LV and RV systolic and diastolic function were similar between groups. NH&PI demonstrated reduced peak LV mid circumferential ε and early diastolic SR, as well as reduced global radial ε. There was reduced basal rotation at 25–35% systole, reduced apical rotation at 25–40% and 60–100% systole and reduced twist at 85–95% systole in NH&PI athletes. There were no differences between the two groups in RV wall mechanics. When compared to Caucasian controls, NH&PI rugby players have a greater LV mass, MWT and RWT with concomitant reductions in circumferential and twist mechanics. This data acts to prompt further research in NH&PI athletes.

## Introduction

The ‘Athletes Heart’ (AH) describes cardiac structural and functional adaptations that occur in response to chronic exercise training [1]. Although there is debate related to the magnitude and timing of adaptation, it is apparent that all cardiac chambers become enlarged with concomitant changes in function [2]. This adaptation often creates a challenge for the clinician when the athlete undergoes pre-participation screening as these physiological changes can mimic those seen in conditions that may predispose an athlete to sudden cardiac death (SCD) [2].

Echocardiography is regarded as an important tool in differential diagnosis within athletic populations. Novel techniques such as speckle tracking echocardiography facilitates the assessment of global and regional cardiac mechanics to determine strain (ε), strain rate (SR) and twist data in the AH [3, 4]. The application of this technique aids the conventional assessment of cardiac function and has been employed in the differential diagnosis of physiological and pathological adaptation [4].

Current clinical criteria to distinguish AH from inherited cardiomyopathies are largely derived from Caucasian athletes [2]. More recently, studies have attempted to explore the impact of ethnicity upon the AH phenotype, with greater LV wall thicknesses observed in African and Afro-Caribbean athletes compared to Caucasian athletes [5–7]. These structural differences exacerbate the challenges in the differential diagnosis in diverse multi-ethnic sporting populations. In addition, there are limited data on right ventricular (RV) structure and function in different ethnic groups [8] with no data on the left or right heart of athletes from a Native Hawaiian and Pacific Island (NH&PI) origin.

Rugby Football League (RFL) athletes provide an ideal model for assessment of the AH due to moderate dynamic and static components of the sport [9]. The recent occurrence of high profile events of SCD within the sport suggests the need to investigate this population. RFL athletes are ethnically diverse, specifically with a high number of participants from the Native Hawaiian and Pacific Island (NH&PI) ethnic group.

The aim of this exploratory study is to investigate the AH phenotype in NH&PI RFL athletes. Specifically, (1) to describe conventional echocardiographic indices of LV and RV structure and function in NH&PI RFL players and matched RFL Caucasian controls (CC) and (2) to demonstrate LV and RV mechanics in these populations.

## Methods

### Study design and population

Twenty-one male RFL athletes of NH&PI ethnicity (mean age ± SD, 27 ± 3 years) were recruited into this cross-sectional study. Results were compared to a group of age-matched male elite RFL athletes of Caucasian ethnicity (mean age ± SD, 25 ± 4 years). All participants were free of known cardiovascular disease and avoided alcohol and caffeine consumption 24 h prior to data collection, whilst refraining from training for at least 6 h prior to the examinations. Ethics approval was granted by the Ethics Committee of Liverpool John Moores University.

### Procedures

Blood pressure (BP), body mass (Seca 217, Hannover, Germany) and height (Seca Supra 719, Hannover, Germany) were recorded. Body surface area (BSA) was calculated as previously described [10]. All participants completed a health questionnaire to exclude cardiovascular symptoms, family history of sudden cardiac death and any other cardiovascular history and/or abnormalities. Ethnicity was self-reported by the athletes using the health questionnaire and subsequently recorded for future reference. A 12-lead ECG (CardioExpress SL6, Spacelab Healthcare, Washington, USA) was used to determine training and non-training related abnormalities [11].

A standard echocardiogram was undertaken by a single experienced sonographer using a commercially available ultrasound system (Vivid Q, GE Healthcare, Horten, Norway) and a 1.5-4 MHz phased array transducer. All images were acquired in accordance with the American Society of Echocardiography (ASE) guidelines [12]. Images were stored in a raw DICOM format and exported to an offline workstation (EchoPac version 7.0, GE Healthcare, Horten, Norway) for subsequent analysis. All data was analysed by a single experienced sonographer. A minimum of three cardiac cycles were averaged for all acquisitions.

### Conventional 2D echocardiography

Standard measurements were made in accordance with ASE guidelines [12]. LV linear dimensions (LVIDd and LVIDs) allowed the calculation of LV mass using the ASE corrected equation. To provide a comprehensive assessment of LV wall thickness, eight measurements were made from a parasternal short axis orientation at basal and mid-levels from the anteroseptum, infero-septum, posterior wall and lateral wall [13]. Mean wall thickness (MWT) was calculated as an average of all eight segments. Relative wall thickness (RWT) was calculated using the formula [(RWT = IVSWTd + PWTd)/LVIDd], where IVSWTd denotes diastolic basal interventricular septal wall thickness and PWTd denotes diastolic basal posterior wall thickness. LV end diastolic volume (LVEDV), LV end systolic volume (LVESV), stroke volume (SV) and ejection fraction (EF) were calculated using a Simpson’s biplane method. Pulsed-wave Tissue Doppler Imaging (TDI) assessed the septum and lateral wall for systolic (S′), early (E′) and late (A′) diastolic velocities.

The RV outflow tract (RVOT) was measured at three locations (RVOT_plax_, RVOT_1_ and RVOT_2_). The RV inflow was measured from a modified apical four chamber orientation and included the base (RVD_1_), the mid-level (RVD_2_) and the length (RVD_3_). RV diastolic area (RVDa) and RV systolic area (RVSa) were measured and RV fractional area change was calculated (RVFAC). RV wall thickness (RVWT) was measured from a sub-costal approach. Tricuspid annular plane systolic excursion (TAPSE) was measured and TDI allowed for assessment of RV lateral S′, E′ and A′.

All structural indices were scaled allometrically to BSA based on the principle of geometrical similarity [14, 15]. Linear dimensions were scaled to BSA^0.5^, areas directly to BSA and volumes to BSA^1.5^, with LV mass also scaled to height^2.7^ [16].

### Myocardial speckle tracking

All images were acquired at a frame rate maintained between 40 and 90 frames per second, and settings were adjusted to provide optimal endocardial delineation. Images were analysed offline (EchoPac, Version 7.0, GE Healthcare, Horten, Norway). The ROI was adjusted to encompass the whole myocardium.

LV longitudinal ε and SR were assessed using the apical four-chamber, two-chamber and three-chamber views, providing a global value based on the average of 18 segments. The parasternal short-axis view allowed the assessment of LV basal circumferential and radial ε and SR. Peak values were averaged from six myocardial segments assessed at the level of the mitral valve, whilst the level at the papillary muscle provided the same data at mid-level. The average global circumferential and radial ε and SR was calculated from basal and mid values and hence 12 myocardial segments.

Basal rotation was measured using the circumference of the LV at the mitral valve level. A parasternal short-axis view at the level of the apex, defined as the level just above the point of systolic cavity obliteration, was used to assess apical rotation. LV twist was calculated as the net difference between apical and basal rotation.

RV longitudinal ε and SR were assessed using a modified four-chamber view. RV global longitudinal ε and SR were averaged from three myocardial segments with the ROI restricted to the lateral wall only.

### Temporal analysis

Raw data from STE assessment was exported into a spreadsheet and processed using cubic spline interpolation. Temporal data across systole and diastole in 5% increments of absolute cardiac cycle were defined. Average ε and SR values across each 5% increment were used to create temporal curves and graphs.

### Statistical analysis

Study data were collected and managed using REDCap electronic data capture tools hosted at Liverpool John Moores University [17]. All echocardiographic data are presented as mean ± SD. Statistical analyses were performed using SPSS Version 23.0 for Windows (SPSS, Chicago, Illinois, USA). All variables, including ε and SR at each 5% increment time point, were analysed for normality using a Shapiro–Wilk test. All normally distributed variables were analysed between groups using an independent T test, with non-normally distributed variables analysed using a non-parametric Mann–Whitney U test. ε and SR 5% increments for systole and diastole were analysed for group by time interactions using a Two-Way ANOVA. The p value of < 0.05 was considered statistically significant.

## Results

Both the NH&PI and CC were similar (p > 0.05) in height (1.83 ± 0.06 and 1.83 ± 0.05 m), BSA (2.29 ± 0.16 and 2.21 ± 0.12 kg/m^2^), heart rate (57 ± 12 and 55 ± 9 bpm), cardiac output (5.26 ± 1.09 and 4.8 ± 0.66 L/min), systolic BP (131 ± 10 and 129 ± 8 mmHg), diastolic BP (70 ± 7 and 69 ± 9 mmHg), and training hours (23 ± 10 and 23 ± 7 h/week). NH&PI athletes had a significantly larger body mass than the CC (103 ± 11 and 96 ± 8 kg). There were no differences between groups for ECG criteria. Normal ECG findings included sinus bradycardia (NH&PI 57%, CC 76%), sinus arrhythmia (NH&PI 10%, CC 10%), 1st degree AV block (NH&PI 19% and CC 5%), partial RBBB (NH&PI 14%, CC 10%), early repolarisation (NH&PI 95%, CC 67%) and isolated criteria for LVH (NH&PI 14%, CC 19%) [11]. T wave inversion > 2 mm in the anterior leads was found in one athlete from each ethnic group. On subsequent analysis, these findings were considered physiological in both cases as determined by echocardiography and cardiac magnetic resonance imaging.

### Conventional structure and function

**Fig. 1:**
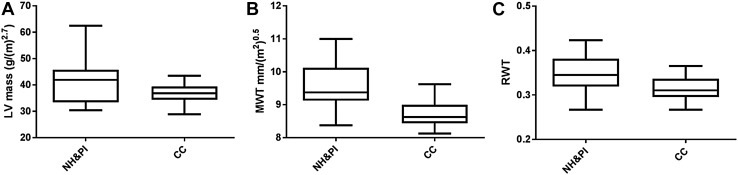
Box plot demonstrating, **a** LV mass, **b** mean wall thickness, **c** relative wall thickness between NH&PI and CC

Structural and standard functional indices of the LV and RV are presented in Table 1 and Fig. 1. LV mass, MWT, RWT and RVWT were significantly larger in NH&PI compared with CC. There were no between-group differences in LV and RV cavity dimensions and all standard systolic and diastolic functional indices.

**Table 1:** Echocardiographic parameters of the LV and RV

Parameter	Mean ± SD		p value
	NH&PI	CC	
Left ventricle			
IVSWTd (mm)	10 ± 1	9 ± 1	0.006*
PWTd (mm)	10 ± 1	9 ± 1	0.012*
LVIDd [mm/(m^2^)^0.5^]	37 ± 3	38 ± 2	0.229
LVIDs [mm/(m^2^)^0.5^]	26 ± 2	26 ± 2	0.878
LV mass [g/(m^2^)^2.7^]	42 ± 9	37 ± 4	0.017*
MWT (mm)	10 ± 0.7	9 ± 0.4	< 0.001*
RWT	0.35 ± 0.04	0.31 ± 0.03	0.003*
LVEDV [ml/(m^2^)^1.5^]	47 ± 8	47 ± 7	0.873
LVESV [ml/(m^2^)^1.5^]	19 ± 4	20 ± 4	0.566
LV SV (ml)	94 ± 19	89 ± 13	0.27
LV EF (%)	59 ± 4	58 ± 5	0.515
Septal S′ (cm/s)	9 ± 1.3	9.3 ± 1.1	0.378
Septal E′ (cm/s)	11.7 ± 1.5	12.5 ± 2.4	0.191
Septal A′ (cm/s)	7.5 ± 1.6	7.8 ± 1.6	0.575
Lateral S′ (cm/s)	11.5 ± 2.9	11.6 ± 2.4	0.861
Lateral E′ (cm/s)	17 ± 3.4	18.5 ± 2.7	0.108
Lateral A′ (cm/s)	7.7 ± 2.7	6.6 ± 2	0.120
Right ventricle			
RVOT_PLAX_ index [mm/(m^2^)^0.5^]	23 ± 2	23 ± 2	0.981
RVOT_1_ index [mm/(m^2^)^0.5^]	23 ± 3	24 ± 3	0.476
RVOT_2_ index [mm/(m^2^)^0.5^]	18 ± 2	19 ± 3	0.212
RVD_1_ index [mm/(m^2^)^0.5^]	29 ± 2	30 ± 4	0.229
RVD_2_ index [mm/(m^2^)^0.5^]	23 ± 3	21 ± 3	0.085
RVD_3_ index [mm/(m^2^)^0.5^]	56 ± 13	62 ± 6	0.054
RVDa Index (cm/m^2^)	13 ± 2	13 ± 2	0.399
RVSa Index (cm/m^2^)	7 ± 1	7 ± 2	0.809
RVWT (mm)	5 ± 1	4 ± 1	0.016*
TAPSE (mm)	24 ± 4	25 ± 4	0.742
RVFAC (%)	47 ± 6	49 ± 7.2	0.365
RV S′ (cm/s)	15 ± 2	14 ± 2	0.442
RV E′ (cm/s)	14 ± 3	15 ± 3	0.27
RV A′ (cm/s)	11 ± 2	10 ± 3	0.161

*LV* left ventricle, *RV* right ventricle, *NH&PI* Native Hawaiian & Pacific Islanders, *CC* Caucasian Controls, *d* at end-diastole, *s* at end-systole, *IVSWT* interventricular septal wall thickness, *PWT* posterior wall thickness, *LVID LV* internal diameter, *MWT* mean wall thickness, *RWT* relative wall thickness, *LVEDV LV* end-diastolic volume, *LVESV LV* end-systolic volume, *SV* stroke volume, *EF* ejection fraction, *RVOT* RV outflow tract, *RVD* RV dimension, *RVDa* RV area at end-diastole, *RVSa* RV area at end-systole, *RVWT* RV wall thickness, *TAPSE* tricuspid annular plane systolic excursion, *RVFAC* RV fractional area change

*p < 0.05 NH&PI versus CC

### Mechanics

#### Peak data

Peak LV and RV ε and SR data are presented in Table 2. LV mid circumferential peak ε, LV mid circumferential SRE, LV global circumferential SRE and LV global radial SRE were significantly lower in the NH&PI compared to the CC. All other peak LV mechanical data, including twist indices and peak RV mechanical data, were similar between groups.

**Table 2:** LV and RV ε and SR

Parameter	Mean ± SD		p value
	NH&PI	CC	
LV longitudinal			
ε (%)	− 19 ± 2	− 19 ± 2	0.897
Time to peak ε (s)	0.38 ± 0.03	0.37 ± 0.03	0.325
SRS (S^−1^)	− 0.93 ± 0.09	− 0.95 ± 0.1	0.549
SRE (S^−1^)	1.29 ± 0.18	1.36 ± 0.18	0.229
SRA (S^−1^)	0.67 ± 0.16	0.62 ± 0.14	0.294
LV circumferential			
ε (%)	− 20 ± 3	− 21 ± 2	0.094
Time to peak ε (s)	0.38 ± 0.03	0.37 ± 0.03	0.785
SRS (S^−1^)	− 1.09 ± 0.2	− 1.17 ± 0.12	0.154
SRE (S^−1^)	1.52 ± 0.35	1.8 ± 0.37	0.023*
SRA (S^−1^)	0.43 ± 0.13	0.45 ± 0.09	0.659
ε mid (%)	− 20 ± 3	− 21 ± 2	0.029*
Mid SRS (S^−1^)	− 1.11 ± 0.21	− 1.18 ± 0.16	0.261
Mid SRE (S^−1^)	1.44 ± 0.34	1.7 ± 0.38	0.019*
Mid SRA (S^−1^)	0.51 ± 0.12	0.5 ± 0.14	0.973
ε basal (%)	− 20 ± 4	− 21 ± 3	0.347
Basal SRS (S^−1^)	− 1.14 ± 0.2	− 1.21 ± 0.14	0.222
Basal SRE (S^−1^)	1.69 ± 0.51	1.91 ± 0.45	0.153
Basal SRA (S^−1^)	0.4 ± 0.21	0.39 ± 0.09	0.703
LV radial			
ε (%)	43 ± 14	50 ± 12	0.112
Time to peak ε (s)	0.41 ± 0.04	0.40 ± 0.5	0.697
SRS (S^−1^)	1.57 ± 0.31	1.66 ± 0.34	0.371
SRE (S^−1^)	− 1.79 ± 0.34	− 2.1 ± 0.43	0.013*
SRA (S^−1^)	− 1.01 ± 0.56	− 0.94 ± 0.33	0.87
ε mid (%)	43 ± 18	51 ± 16	0.13
Mid SRS (S^−1^)	1.71 ± 0.47	1.68 ± 0.37	0.825
Mid SRE (S^−1^)	− 1.99 ± 0.57	− 2.33 ± 0.81	0.122
Mid SRA (S^−1^)	− 1.12 ± 0.66	− 0.96 ± 0.45	0.367
ε basal (%)	43 ± 4	58 ± 3	0.195
Basal SRS (S^−1^)	1.81 ± 0.46	1.93 ± 0.55	0.434
Basal SRE (S^−1^)	− 1.6 ± 0.53	− 1.87 ± 0.53	0.099
Basal SRA (S^−1^)	− 0.91 ± 0.57	− 0.93 ± 0.4	0.917
LV rotation			
Apical rotation (°)	7.1 ± 3.8	9.3 ± 3.6	0.054
Basal rotation (°)	− 6.9 ± 2.6	− 6.7 ± 3.5	0.873
Twist (°)	13.4 ± 4.6	15.6 ± 5.1	0.159
RV longitudinal			
ε (%)	− 27 ± 3	− 27 ± 4	0.857
Time to peak ε (s)	0.39 ± 0.03	0.38 ± 0.03	0.252
SRS (S^−1^)	− 1.24 ± 0.2	− 1.31 ± 0.21	0.266
SRE (S^−1^)	1.60 ± 0.35	1.56 ± 0.36	0.771
SRA (S^−1^)	0.82 ± 0.26	0.84 ± 0.28	0.642

*LV* left ventricle, *RV* right ventricle, *ε* strain, *SR* strain rate, *NH&PI* Native Hawaiian & Pacific Islanders, *CC* caucasian controls

*p < 0.05 NH&PI versus CC

### Temporal analysis

Significant group by time interactions (p < 0.05) were present for mid circumferential strain in systole, with systolic twist producing a trend towards significance (p = 0.058). There were also significant interactions during diastole for global and mid circumferential, and mid radial SR, as well as apical twist.

**Fig. 2:**
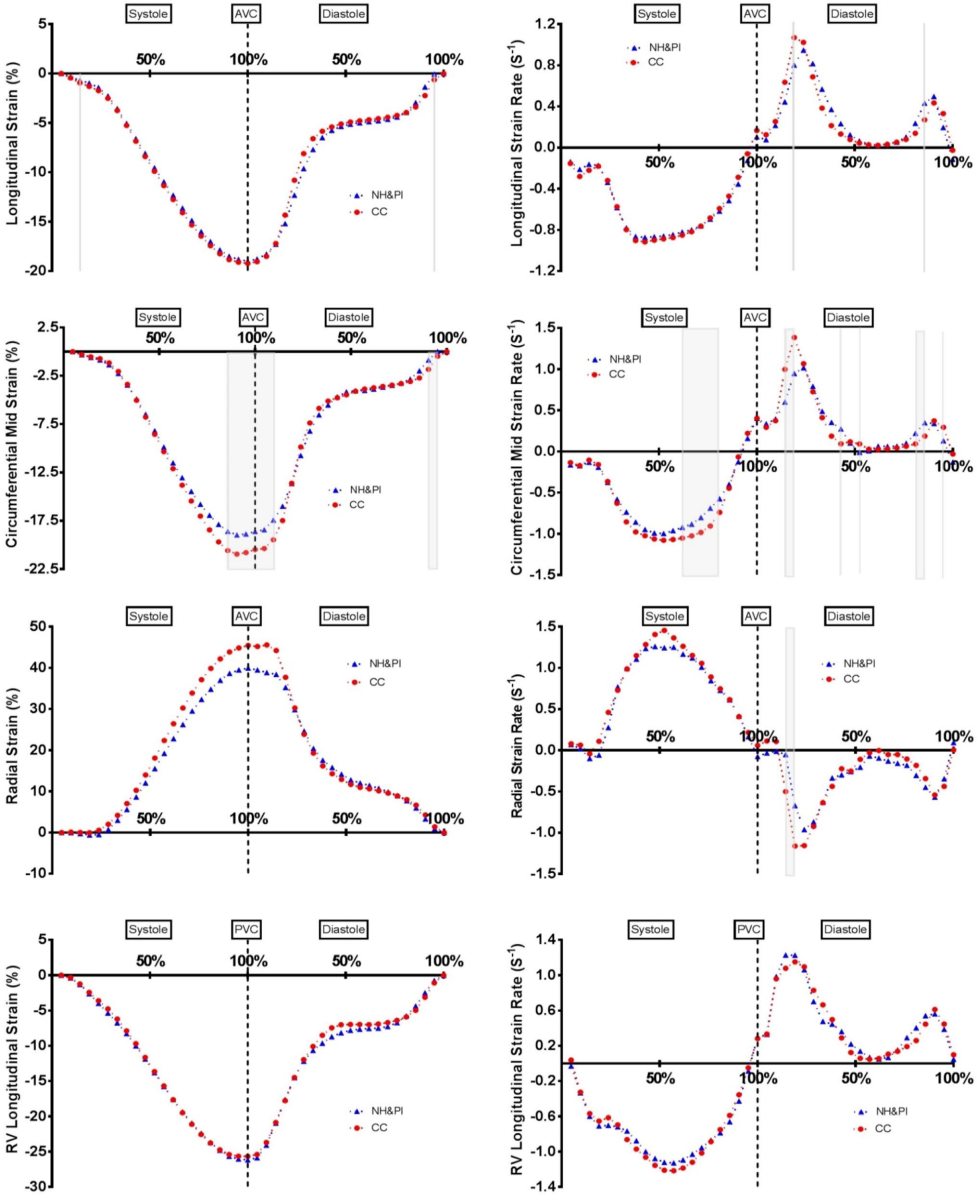
Left ventricular longitudinal, mid circumferential, radial and right ventricular longitudinal ε and strain rate—grey area denotes statistical significance (p < 0.05). *AVC* Aortic valve closure, *PVC* pulmonary valve closure

**Fig. 3:**
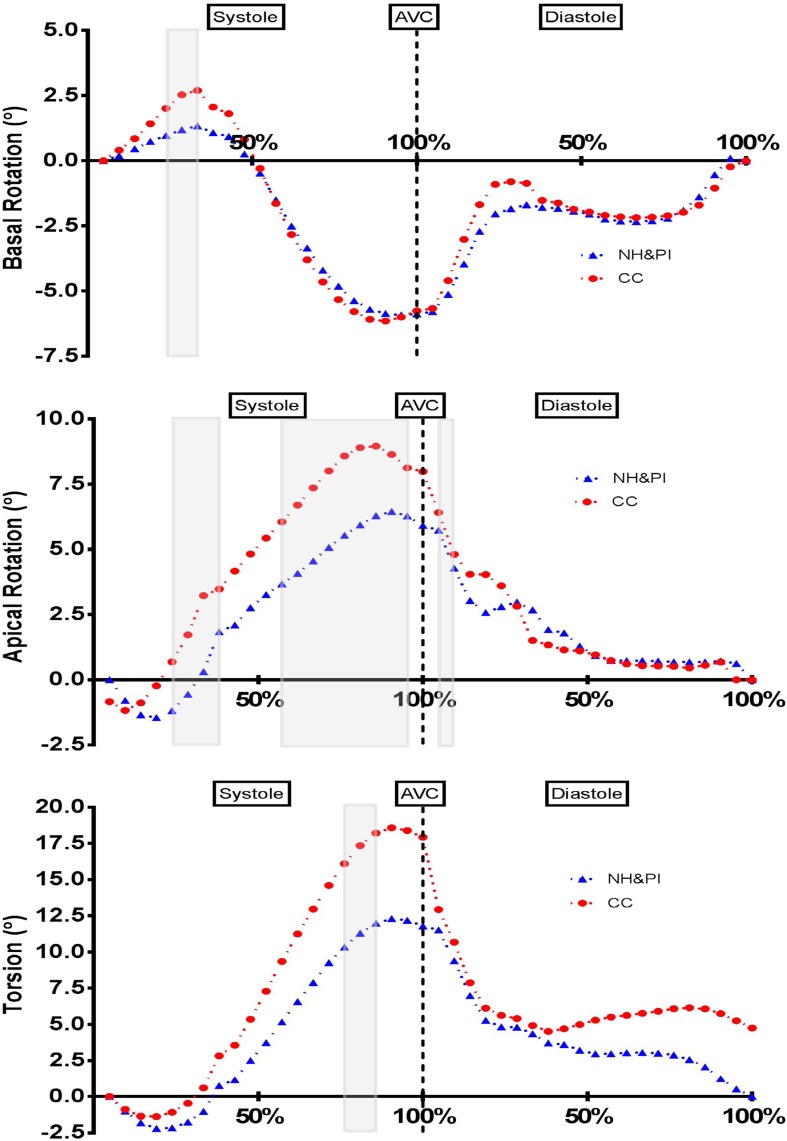
Left ventricular basal rotation, apical rotation and torsion—grey area denotes statistical significance (p < 0.05)

Individual systolic and diastolic 5% increment analysis demonstrated global circumferential ε (late diastole), mid circumferential ε (late systole through to early diastole and late diastole) and mid radial ε (early systole) as lower in NH&PI athletes (see Fig. 2). Likewise, there was evidence of reduced SR in NH&PI athletes (global longitudinal SR in early diastole; global, mid and basal circumferential SR in early diastole; global and mid radial SR in early diastole; global circumferential SR in late diastole). There was also evidence of reduced basal rotation at 25–35% systole, reduced apical rotation at 25–40% and 60–100% systole and reduced twist at 85–95% systole in NH&PI athletes (see Fig. 3). On the contrary, there were no temporal differences in any RV mechanical data (see Fig. 2).

## Discussion

The main findings of this exploratory study are (1) NH&PI athletes have larger LVM, MWT, RWT and RVWT compared to CC with no between-group differences in standard LV and RV conventional functional indices and (2) there is evidence of reduced peak and temporal LV mechanics but no difference in RV mechanics in NH&PI athletes compared to CC.

### Structural adaptation

In the current study the NH&PI demonstrated larger LV mass and thicker LV walls than CC with no significant differences in LV cavity dimensions. The combination of larger LV mass, thicker LV walls and similar LV cavity dimensions in NH&PI meant that although none of the athletes met the absolute criteria for concentric remodelling, they had values closer to the cut-off [12]. A similar pattern of increased wall thicknesses in African/Afro-Caribbean athletes compared to other ethnic backgrounds has previously been demonstrated [5, 6]. Although previous studies have described resting BP differences between ethnicities [18], our study reports similar resting BP between the NH&PI and CC. Therefore, the known notion that haemodynamic stress in the form of an increased BP increases ventricular afterload causing adaptation in ventricular wall thickness does not explain the current differences found between the NH&PI and CC. Therefore, we suggest a possible difference in the response of the LV to alterations in cardiac load during exercise training in NH&PI athletes, when compared to a CC population, which could follow a similar pattern to their African counterparts [5, 6]. It is important to note that currently there is no data pertaining to the acute cardiac responses to exercise in the NH&PI population, and we believe this should be further investigated. This phenomenon has also been observed in a pathological response to pressure overload in hypertensive disease [19] and therefore further work should aim to establish any possible implications to the NH&PI population.

RV structure in NH&PI athletes followed a similar pattern to the phenotype seen in the LV with increased RVWT but similar RV cavity dimensions. Zaidi et al. [8] investigated the RV in athletes of African and Afro-Caribbean origin compared to a CC athletic population. RVOT values were smaller in the African athletic population compared to CC but with a trend towards larger RVWT (p = 0.05). Our findings in NH&PI suggest that these athletes have a similar adaptive response to the exercise stimulus in the RV which may be explained through evidence of a concomitant/balanced increase in RV and LV adaptations in respect to mass and volume [20].

This type of AH morphology may exacerbate the difficulty in distinguishing physiology from pathology in NH&PI athletes [21]. That aside, it is important to note that none of the athletes in this study had absolute LV wall thicknesses greater than 12 mm. However, RFL is a moderate static and dynamic sport and it is speculative to suggest that NH&PI athletes engaged in other sports [9] may present with even greater wall thicknesses compared to their Caucasian counterparts [22].

### Functional adaptation

Although systolic function as determined by conventional imaging was similar between ethnic groups, reduced circumferential and radial mechanics in systole and diastole were observed in NH&PI athletes. It is difficult to directly attribute these findings to the differences in LV geometry but reduced peak circumferential ε has previously been reported in body builders who exhibited increased LV mass and wall thickness compared to a marathon runner cohort [23]. It may be that adequate LV forces are generated with lower circumferential contribution. The McIver Townsend hypothesis [24] may partially explain this phenomenon. The hypothesis states that alterations in mechanical function are a product of an increase in LV hypertrophy (LVH). Essentially, radial displacement is maintained even in the presence of reduced longitudinal function and non-compensatory circumferential function. LVH, and therefore myocardial muscle volume, leads to an increased endocardial displacement for any given level of deformation. These findings need to be reproduced in a much larger NH&PI population but these exploratory findings are important from both a physiological and clinical perspective.

In addition to circumferential mechanics we also observed significantly reduced apical and basal rotation in early systole consistent with an extended pre-twist period in the NH&PI. A reduction in apical rotation throughout systole resulted in lower twist mechanics. It could be suggested that the alteration in early diastolic SR in the NH&PI could be a consequence of the reduced apical rotation observed. Apical twist is considered an important component of LV function to store additional potential energy that is released to increase early diastolic suction [25]. This “recoil” causes a rapid reduction of LV pressure leading to early diastolic filling [25]. A reduced store of potential energy could attenuate the “recoil” relaxation of the ventricle. A previous deformation study [26] also highlighted that apical rotation is the primary determinant of peak systolic LV twist. It is not possible from the current study to conclude as to whether reduced apical rotation is due to a reduced mechanical functionality that could be linked to the increased wall thickness present, as seen in patients with HCM [27] or if it is an amplification of the previously identified adaptive response to training concluded as a “reserve” for the onset of exercise [28]. Therefore, as mentioned previously, further research is needed to evaluate the acute cardiac response to exercise within the NH&PI population.

Our findings of similar RV mechanics in the presence of increased RVWT in NH&PI athletes is reassuring and highlights a similar response to the LV in this ethnic group. It is important to note that it is not possible to accurately assess RV circumferential function and therefore a comprehensive RV functional assessment may provide further insight.

### Limitations

This study is limited by a small sample size and hence we deemed this an exploratory study. However, PostHoc analysis of the MWT demonstrated 100% statistical power, therefore demonstrating adequate statistical power with this sample size.

It is also important to note that we selected our athletes for their sporting discipline, however, these findings cannot be generalizable to athletes of other sports, sex or age. Hence further work to address the multi-factorial nature of the AH alongside ethnic specific adaptation is required.

It is important to note that longitudinal ε is the most commonly used indicator of LV mechanics in practice, with radial and circumferential ε deemed to carry limitations in terms of reproducibility in both physiological and pathological subjects.

## Conclusion

This study demonstrated that athletes of NH&PI ethnicity have greater LV and RV wall thickness and mass compared to their Caucasian counterparts. In addition, there is reduced LV circumferential and twist mechanics that may reflect the differences in LV geometry structural adaptation.

## Figures and Tables

**Fig. 4:**
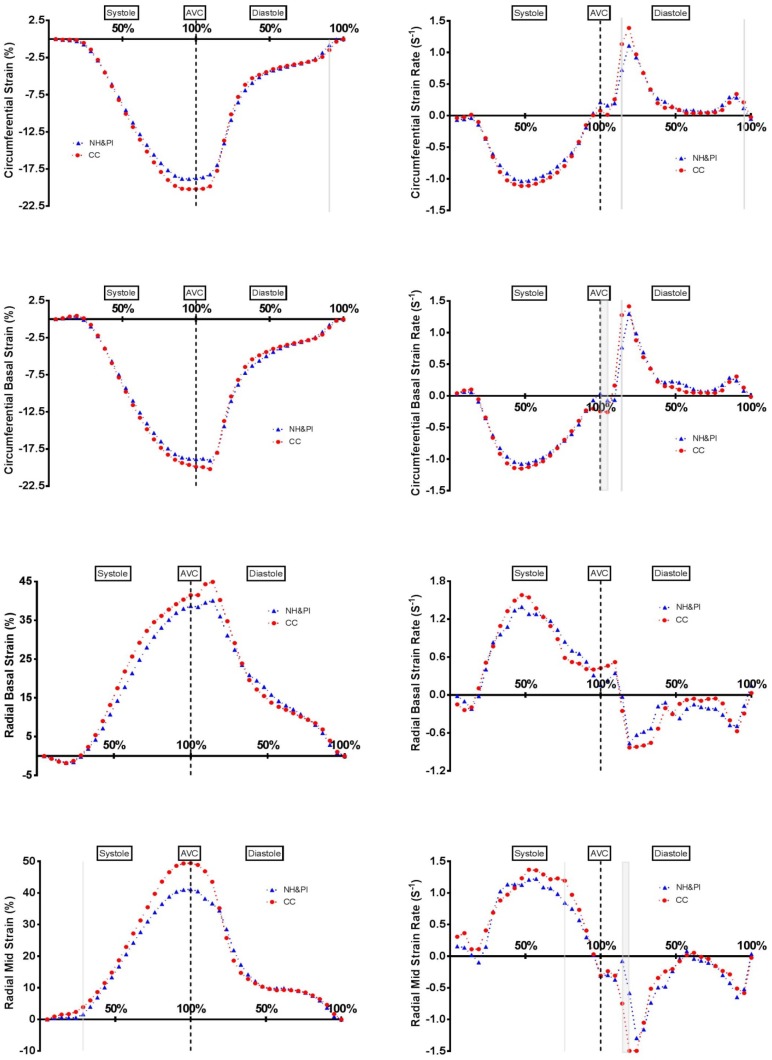
Circumferential and radial mechanics

## Appendix

See Fig. 4.
